# Protein from Meat or Vegetable Sources in Meals Matched for Fiber Content has Similar Effects on Subjective Appetite Sensations and Energy Intake—A Randomized Acute Cross-Over Meal Test Study

**DOI:** 10.3390/nu10010096

**Published:** 2018-01-16

**Authors:** Lone V. Nielsen, Marlene D. Kristensen, Lars Klingenberg, Christian Ritz, Anita Belza, Arne Astrup, Anne Raben

**Affiliations:** Department of Nutrition, Exercise and Sports, Faculty of Science, University of Copenhagen, Rolighedsvej 30, DK-1958 Frederiksberg, Denmark; marlenedkristensen@gmail.com (M.D.K.); lakl@nexs.ku.dk (L.K.); ritz@nexs.ku.dk (C.R.); anbelsp@gmail.com (A.B.); ast@nexs.ku.dk (A.A.); ara@nexs.ku.dk (A.R.)

**Keywords:** animal protein, fava beans, split pea, veal, pork, bean, satiety, egg

## Abstract

Higher-protein meals decrease hunger and increase satiety compared to lower-protein meals. However, no consensus exists about the different effects of animal and vegetable proteins on appetite. We investigated how a meal based on vegetable protein (fava beans/split peas) affected ad libitum energy intake and appetite sensations, compared to macronutrient-balanced, iso-caloric meals based on animal protein (veal/pork or eggs). Thirty-five healthy men were enrolled in this acute cross-over study. On each test day, participants were presented with one of four test meals (~3550 kilojoules (kJ) 19% of energy from protein), based on fava beans/split peas (28.5 g fiber), pork/veal or eggs supplemented with pea fiber to control for fiber content (28.5 g fiber), or eggs without supplementation of fiber (6.0 g fiber). Subjective appetite sensations were recorded at baseline and every half hour until the ad libitum meal three hours later. There were no differences in ad libitum energy intake across test meals (*p* > 0.05). Further, no differences were found across meals for hunger, satiety, fullness, prospective food consumption, or composite appetite score (all *p* > 0.05). Iso-caloric, macronutrient-balanced, fiber-matched meals based on vegetable protein (fava beans/split peas) or animal protein (veal/pork or eggs) had similar effects on ad libitum energy intake and appetite sensations.

## 1. Introduction

In recent decades, high protein diets have attracted considerable attention and substantial evidence now supports improved weight loss and weight loss maintenance after consumption of high-protein diets [1]. These effects can be, at least partly, attributed to the highly satiating and thermogenic effect of proteins [2,3]. 

Health authorities recommend an increase in dietary protein from plant-based sources, at the expense of animal protein, since plant-based proteins sources have low energy density, high fiber content, and their production is associated with a lower emission of greenhouse gases [4,5]. Lately, several studies have investigated the different effects of proteins from plant and animal sources on appetite and energy intake [6,7,8]. Kehlet et al. found no difference in appetite sensations and energy intake when soy-based vegetable patties were compared to fiber-matched meatballs in an acute meal test study [6]. Douglas et al. also found no differences in hunger, fullness, or energy intake when comparing a high-protein soy-based lunch with a macronutrient- and fiber-matched high-protein beef-based lunch [7]. However, they investigated meals with a fairly large protein content (33 E%), which could have masked potential differences between the protein sources. We have previously shown that a meal with vegetable protein from fava beans and split peas increased satiety and decreased hunger and ad libitum energy intake compared to a meal with animal protein from pork and veal [8]. That study was, however, not able to demonstrate that vegetable protein from legumes per se was more satiating than animal protein, as the study allowed natural differences in dietary fiber content across the meals [8]. 

Only a few studies have investigated how egg affects appetite compared to other protein sources. Pal and Ellis found whey protein to be superior in stimulating satiety compared to tuna, egg, and turkey when served as a macronutrient-balanced liquid breakfast meal. Also, tuna stimulated satiety to a greater extent than egg or turkey [9]. Additionally, Anderson et al. observed a higher energy intake after a liquid preload of egg albumin compared to a preload with soy or whey protein [10]. However, it is not evident if the effects of protein from eggs are similar when ingested as solid foods in whole meals [11]. 

Studies that compare animal and vegetable protein sources using whole meals, controlling for macronutrient content and fiber, are limited. Therefore, the aim of the present study was to investigate if a meal based on vegetable protein from fava beans and split peas would reduce ad libitum energy intake and subjective appetite sensations compared to iso-caloric, macronutrient-balanced, fiber-matched meals based on animal protein from pork and veal or eggs. The role of a similar egg meal without supplemented fiber was also investigated.

## 2. Materials and Methods 

### 2.1. Subjects

Healthy, weight stable (maximum ± 3 kg in the previous two months), non-smoking men, aged 18–50 years, with a body mass index (BMI) of 18.5–30.0 kg/m^2^, were recruited for the study. Exclusion criteria included: mental disorders, metabolic diseases, systematic or/and chronic infections, use of medication that is considered to affect appetite, food allergies of relevance to the test meals, vegetarians, vigorous physical activity of more than 10 h per week, current or previous drug abuse, alcohol intake above 14 units per week, or simultaneous participation in other studies. The participants were individually interviewed reading the inclusion and exclusion criteria at a screening visit, where they also completed the three-factor eating questionnaire [12]. Height was measured on a wall-mounted stadiometer with 0.5-centimeter accuracy (HeightTronic235, Quick Medical, Issaquah, WA, United States of America). Body weight was recorded to the nearest 0.05 kg on a decimal scale (LindelTronic 8000, Samhall Lavi AB, Kristianstad, Sweden). 

All subjects gave their written informed consent for inclusion before they participated in the study. The study was conducted in accordance with the Declaration of Helsinki of 1975, revised in 1983, and was registered at clinicaltrials.gov (NCT01616251).

### 2.2. Experimental Design

This study was performed at the Department of Nutrition, Exercise and Sports, University of Copenhagen, Denmark between August and December 2012, and was conducted as a single-blinded randomized, four-condition, cross-over meal test study. On the four test days, the participants arrived in the laboratory at the same time in the morning (at 8:00 a.m. or 8:30 a.m.). Upon arrival, participants were required to attempt to void and body weight was recorded. After a 10-min rest, the first visual analogue scales (VAS) were provided to assess subjective appetite sensations in the fasting state. Participants were then served one of the four test meals (time 0), which they ingested during the next 15 min. Subsequently, the participants filled in a VAS on subjective appetite sensations, and for the palatability of the test meal. Assessment of subjective appetite sensations was repeated at 30, 60, 90, 120, 150, and 180 min after consumption of the meal. At 180 min, the participant completed an additional VAS regarding gastro-intestinal feelings. Three hours after the test meal an ad libitum lunch meal was served. After the ad libitum meal, the participants were asked to complete the last VAS regarding appetite sensations and palatability. To validate possible compensatory energy intake, participants were instructed to complete a weighed food record from the time they were discharged from the laboratory until midnight. On the first test day, the participants were carefully instructed on how to fill in the records. A timeline of the test day measurements is shown in Table 1. Participants were placed in rooms with a maximum of three participants in each, and shielding was placed so they were not able to see each other. During the test day, participants were told to remain awake and were not allowed to talk or communicate with each other. They were not allowed to use mobile phones, the Internet, listen to music, watch films, or play computer games. They were allowed to read and work on their laptops, unless it was related to food or health.

### 2.3. Standardization

Participants were instructed to eat a standardized 4 MJ dinner (a paprika dish with pork and rice: 17 E% protein, 50 E% carbohydrate and 33 E% fat), provided as a frozen product beforehand, between 7.00 p.m. and 8.00 p.m. on the evening prior to the test days and to fast from 8.00 p.m. onward. During the fast, participants were allowed to drink 500 mL tap water, with a maximum of 250 mL allowed on the morning of the test days. Furthermore, the participants were told to abstain from alcohol, medicine, and vigorous physical activity 24 h prior to the test days. The test days were separated by a washout period of seven or more days. 

### 2.4. Test Meals

Test meals were served as breakfast meals and consisted of (1) patties with beans served with mashed split peas; (2) patties with minced pork/veal and pea fiber served with fiber-supplemented mashed potatoes; (3) patties with eggs and pea fiber served with fiber-supplemented mashed potatoes; and (4) patties with eggs served with non-fiber-supplemented mashed potatoes. Additionally, all meals were served with ketchup and water. Water loss during preparation was higher for the bean patties, and therefore extra water was served together with the bean and pea meal (300 mL and 333 mL, respectively). The participants were required to consume the entire meal and to drink the water during the meal. The four test meals were iso-caloric (~3550 kJ) and had a similar macronutrient distribution of 19 E% protein, 53 E% carbohydrate, and 28 E% fat (Table 2). Pictures of the test meals are shown in Figure S1.

To obtain equal fiber content in the three fiber-matched meals, pea fiber (Fibradan, DLG Food A/S, Copenhagen, Denmark) was added to the two fiber-supplemented meals. The pea fiber was produced from the shell of dry yellow and green peas. The product contained cellulose (48.5%), hemicellulose (18%), pectin (14%), lignin (2%), and other compounds (17.5%), total dietary fiber content in the product was 90% (*w*/*w*) [13]. The physiochemical properties of the pea fiber have previously been described [13]. 

### 2.5. Ad Libitum Meal 

The ad libitum lunch meal was pasta bolognaise (556 kJ per 100 g; 15 E% protein, 55 E% carbohydrate, and 30 E% fat). Participants were provided with a pot with 1440 g pasta bolognaise together with 300 mL water and served themselves. They were instructed to eat until they felt comfortably satiated. The pot was weighed before the participants were served the meal. Afterward, the pot was weighed again and the amount ingested was calculated. 

### 2.6. Measurements of Subjective Appetite Sensations and Palatability 

Subjective appetite sensations, well-being, and palatability of the meals were assessed using a 100-mm VAS. At each end of the line, the most positive or most negative ratings were anchored [14]. The VAS questions were combined in a small booklet showing one question at a time. The participant received a total of nine VAS booklets on appetite and well-being during the test day. All booklets included questions regarding satiety, fullness, prospective food consumption (PFC), hunger, thirst, well-being, and subjective sensory-specific desire for sweet, fat, salt, and meat or fish. The composite appetite score was calculated as [satiety + fullness + (100 − PFC) + (100 − hunger)]/4 [15]. The VAS questions on appetite included: (1) How hungry do you feel? Responses could range from ‘I am not hungry at all’ to ‘as hungry as I have ever felt’; (2) How full do you feel? Responses could range from ‘not full at all’ to ‘totally full’; (3) How satisfied do you feel? Responses could range from ‘I am completely empty’ to ‘I cannot eat another bite’; (4) How much do you think you could eat right now? Responses could range from ‘nothing at all’ to ‘a lot’. Well-being was assessed with the question: how comfortable do you feel? Responses could range from ‘really bad’ to ‘really good’. Palatability of the meals was assessed immediately after the participants finished the test meal and again after the ad libitum meal. The VAS question on palatability was: palatability ‘bad’ to ‘good’. 

### 2.7. Gastro-Intestinal Feelings 

The VAS booklet used to assess gastrointestinal feelings included eight yes/no questions. Here, participants answered whether they had experienced acid reflux, nausea, stomach pain, flatulence, heartburn, bloating, rumbling, or diarrhea. If the participants answered yes, they had to rate the intensity of the symptom on a 100 mm scale.

### 2.8. Compensatory Eating

Food records, used to validate possible compensatory eating, were entered into the Danish dietary software program (Dankost 3000^®^ version 07.11.03, Dansk Catering Center, Copenhagen, Denmark) and energy intake during the remainder of each test day was calculated.

### 2.9. Blinding and Randomization 

The different meals were coded with a number from 1 to 4. Only the kitchen staff, who prepared the test meals, knew this combination. The kitchen staff provided the test meals to the investigator with an opaque lid and the investigator was not aware of which meal the participant received on the different test days. Before the study was initiated, a list of combinations of the numbers 1–4 was generated. When participants were found eligible for the study, they were assigned the next available combination of meals by the study coordinator. 

### 2.10. Statistical Power 

The primary endpoint was ad libitum energy intake three hours after the test meals. The sample size calculation was based on results from a previous study [8], which showed that 30 participants had to complete the study to detect a mean difference in ad libitum energy intake of 400 kJ three hours after the test meal, with a standard deviation of 750 kJ (*α* = 0.05, *β* = 0.8).

### 2.11. Statistical Analysis

All statistical analyses were performed using R version 3.1.2 (R Core Team, 2016, Vienna, Austria). Baseline characteristics are presented as mean ± standard deviation (SD). Ad libitum energy intake, palatability, incremental area under the curve (iAUC), and incremental area over the curve (iAOC) were analyzed using linear mixed models with meal as fixed effect and subject as random effect. The models were adjusted for age, BMI, meal order, and visit number. As the test meals had a fixed energy content, an additional model, adjusted for estimated daily energy requirement based on the Harris–Benedict equation and an estimated physical activity level of 1.7, was tested for ad libitum energy intake [16]. iAUC and iAOC were calculated using the trapezoidal method. Repeated measurements of subjective appetite sensations were analyzed using linear mixed models, which included a time–meal interaction. The models were adjusted for visit number, age, BMI, fasting value on the actual test day, and meal order. Additional analyses, adjusted for estimated daily energy requirement, were conducted for satiety, hunger, fullness, and PFC. Overall subject and within-visit subject differences were modeled through random effects. Serial correlation between repeated measurements for the same subject within each visit was modeled, assuming a spatial Gaussian correlation structure with decreasing correlation over time. Post hoc tests were conducted as model-based pairwise comparisons adjusted for multiple testing. Table S1 shows pairwise comparisons for each of the outcomes. Multiplicity adjustment of *p*-values was based on the single-step method [17]. Results are presented as mean ± standard error (SE); graphs are based on raw data presented as mean ± standard error of mean (SEM). Additional sensitivity analyses with adjustment for palatability were conducted. For all models, assumptions about normality and homogeneity of variance were checked graphically using residual plots and normal probability plots. Differences in occurrence of gastrointestinal feelings between meals were evaluated using Fisher’s exact test. *p*-values < 0.05 were considered significant.

## 3. Results

### 3.1. Subjects

Thirty-nine potential male participants attended a screening visit at the university. Thirty-five participants were found eligible and recruited to the study (Table 3).

**Figure 1:**
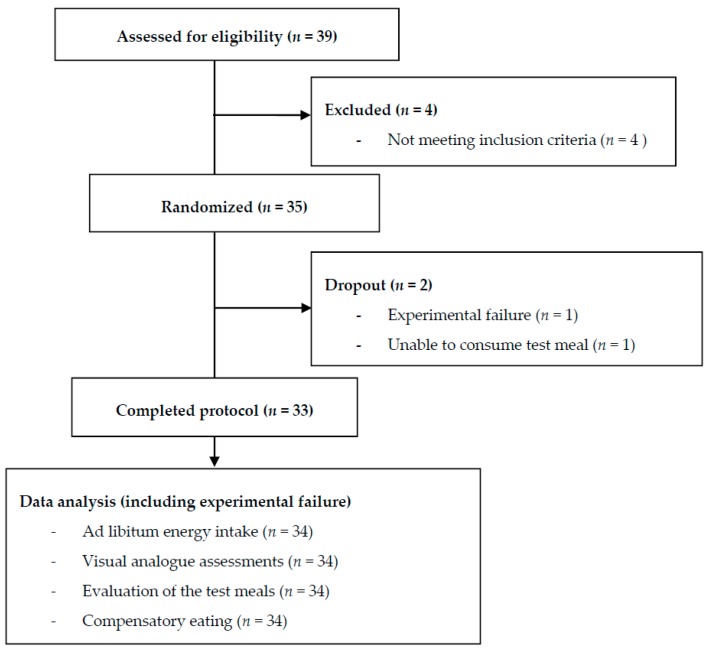
Participant flowchart.

One participant dropped out on the first experimental day, as he was unable to consume the test meal. Another participant received the same test meal twice due to an experimental error. Thus, 33 participants received all four test meals (Figure 1). 

### 3.2. Ad Libitum Energy Intake

**Figure 2:**
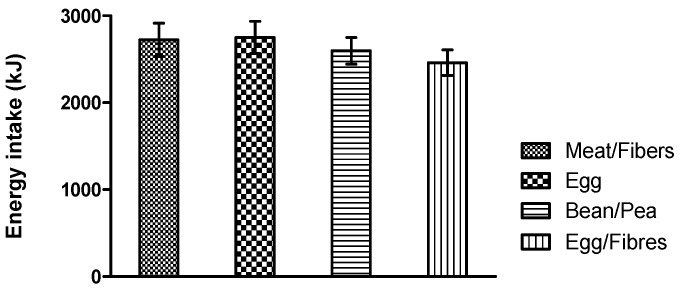
Ad libitum energy intake three hours after the participants received the test meals, analyzed using a mixed linear model including meal as a fixed effect.

No differences were found in energy intake at the ad libitum meal across test meals: meat/fiber 2636 ± 200 kJ (630 ± 48 kcal), egg 2771 ± 201 kJ (662 ± 48 kcal), bean/pea 2608 ± 194 kJ (623 ± 37 kcal), egg/fiber 2513 ± 203 kJ (600 ± 48 kcal), *p* > 0.05 (Figure 2). The level of significance did not change when estimated daily energy requirement was included as a covariate (*p >* 0.05).

### 3.3. Subjective Appetite Sensations, Sensory-Specific Appetite Scores, and Palatability 

Satiety, hunger, fullness, and desire to eat meat or fish, something salty, sweet, or fatty did not differ after the ad libitum meal across test meals. However, the PFC rating was higher following the bean/pea meal compared to the egg/fiber meal (16.1 ± 2.2 mm vs. 11.4 ± 2.3 mm, *p* < 0.05). No differences were found in palatability of the ad libitum meal after the four different test meals (*p* > 0.05).

**Figure 3:**
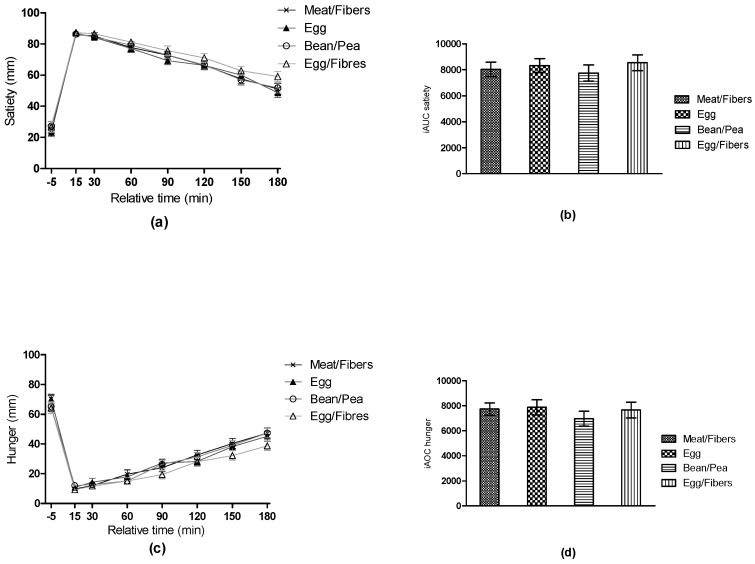
Mean unadjusted three-hour changes in (**a**) satiety, (**c**) hunger, and (**b**) corresponding incremental area under the curve (iAUC) and (**d**) incremental area over the curve (iAOC), respectively. Data are presented as mean ± SEM. Both as repeated measures, analyzed using mixed linear models including a time * meal interaction, and iAUC or iAOC, analyzed using mixed linear models including meal as a fixed effect. No differences were found in satiety and hunger across meals (all *p* > 0.05).

Fasting values of hunger, satiety, fullness, and PFC were not different across the test meals (all, *p >* 0.05). No time * meal interactions or meal effects were found for the postprandial changes in hunger, satiety, fullness, or PFC across test meals (all *p* > 0.05), neither when analyzed separately nor when combined into the composite score (*p* > 0.05). The level of significance did not change when estimated daily energy requirement was included as a covariate (*p >* 0.05). These results remained unchanged when the appetite ratings were analyzed as iAUC or iAOC (all *p* > 0.05) (Figure 3). 

No differences were found in the desire to eat something salty, sweet, or meat or fish across the test meals (all *p* > 0.05). However, a significant meal * time interaction was found for the desire to eat something fatty (*p* < 0.05). Pairwise comparisons, however, did not reveal differences across test meals at any time point (all *p* > 0.05). When analyzing the sensory-specific appetite scores as iAUC, no differences were found across the test meals (all *p* > 0.05). In addition, thirst and well-being were not different across the meals (*p* > 0.05) and no differences existed in gastrointestinal feelings (all *p* > 0.05). 

Palatability was higher for the meat/fiber meal compared to the bean/pea meal (61.3 ± 6.1 mm vs. 39.7 ± 5.7 mm, *p* < 0.001) and the egg/fiber meal (61.3 ± 6.1 mm vs. 45.8 ± 6.2 mm, *p* < 0.01) and for the egg meal compared to the bean/pea meal (54.6 ± 6.1 mm vs. 39.7 ± 5.7 mm, *p* < 0.05). After adjusting the subjective appetite ratings for palatability of the test meals, an overall effect of meal was seen on hunger (*p* < 0.05). Pairwise comparisons revealed that hunger decreased after the egg/fiber meal compared to the meat/fiber meal (*p* < 0.05). 

### 3.4. Compensatory Eating

The completion rate of the food records was 99.2% (134 of 135 potential records were received). No differences were found in energy intake across test meals during the rest of the test day (meat/fiber: 6331 ± 604 kJ (1512 ± 144 kcal); egg: 6009 ± 550 kJ (1435 ± 131 kcal); bean/pea: 6005 ± 567 kJ (1435 ± 135 kcal); and egg/fiber: 5806 ± 619 kJ (1387 ± 148 kcal), (*p* > 0.05). 

## 4. Discussion

In this present study, we did not observe any differences in ad libitum energy intake or appetite sensations between test meals based on vegetable protein compared to fiber-supplemented, macronutrient-balanced, iso-caloric meals based on pork/veal or eggs. Furthermore, no differences were observed when compared to a non-fiber-supplemented egg meal. 

The majority of the previously conducted studies that investigated the effect of vegetable protein compared to animal protein on appetite and energy intake used soy as the vegetable protein source [6,7,18,19]. Douglas et al. investigated how a high-protein lunch with beef affected appetite sensations and energy intake compared to a macronutrient- and fiber-matched high-protein soy lunch [7]. In agreement with our study, no differences in appetite sensations or energy intake between the vegetable and animal protein meal were found. A study by Kehlet et al. supported these findings, as they found a similar effect of fiber-matched meals based on pork or soy on appetite sensations and energy intake [6]. Additionally, a randomized acute meal study by Tan et al. found no differences in satiety levels across iso-caloric high-protein meals with either meat (lean beef and ham), dairy (low-fat milk, cheese, and yogurt), or soy as protein sources [20]. Lang et al. evaluated different protein sources and found no differences in appetite sensations or ad libitum energy intake during the subsequent dinner, after energy-fixed lunch test meals with either, casein, gelatin, or soy [18]. The results from the latter two studies should be interpreted with caution. Tan et al. did not include information regarding fiber whereas the soy meal in the study by Lang et al. contained a higher amount of fiber than the casein and gelatin meal [18,20]. Contrary to these findings, preloads of pasta with tofu or mycoprotein have been observed to reduce energy intake 20 min after consumption compared to a preload of pasta with chicken [19]. These findings are, however, confounded by higher energy content in the meals with tofu and mycoprotein compared to the meal with chicken and higher fiber content in the tofu meal. 

Only a limited number of studies have investigated how bean and pea protein affect appetite sensations and energy intake compared to animal protein. Furthermore, not many studies have used solid and real foods. In a randomized single-blinded cross-over study, no differences in energy intake were found 30 min after a liquid preload of 20 g casein, whey, pea protein, or egg albumin [21]. Lang et al. observed no differences in hunger, satiety, ad libitum energy intake, or 24-h energy intake between lunches enriched with egg white protein (albumin), cow milk protein (casein), gelatin, soy, wheat or pea protein [22]. The study was, however, limited by different energy content and macronutrient composition in the test meals. We previously investigated the effect of meals containing fava beans and split peas as the vegetable protein source compared to pork and veal meat as the animal protein source [8]. In that study, higher satiety and fullness ratings and lower prospective food intake and hunger ratings were found after the fava bean and split pea meal compared to the veal and pork meal. Furthermore, participants had a lower energy intake after the fava bean and split pea meal compared to the veal and pork meal at the ad libitum lunch served three hours after the test meal. In that study, dietary fiber content was not matched but reflected the natural content of the included test foods. Dietary fiber is known to influence appetite [23,24], in this study we matched the fiber content of the meals. This is probably the main reason for the different outcomes of the two studies.

Few studies have investigated the effect of egg on appetite and energy intake compared to other protein sources. Our results indicated that egg has similar effects on appetite as meat (pork and veal) and vegetable proteins (fava beans and split peas). This seems inconsistent with some results from previous studies [9,10]. Yet, these studies served the meals as liquid breakfasts or liquid preloads. Liquid meals are known to have less effect on satiety compared to solid meals and this could, therefore, have influenced the results [25]. Bayham et al. compared an egg breakfast to an energy density and macronutrient-matched cereal breakfast and observed no difference in energy intake at a subsequent lunch served three hours after the test meal. However, the participants felt more full 30 min after the egg breakfast compared to the cereal breakfast [11]. These results are, at least partly, in agreement with our findings.

Since the greenhouse gas emissions associated with meat production are high compared to vegetable production, a reduction in consumption of protein from animal sources is highly recommended [4,5]. In our study, we found a comparable effect on ad libitum energy intake and subjective appetite sensations after the vegetable protein meal that included fava beans and split peas and the fiber-supplemented meals with eggs or veal and pork, and the non-fiber-supplemented egg meal. This demonstrates that legumes are a good alternative to meat with regard to the effect on appetite. Additionally, intake of legumes may also be beneficial for weight management. A recent meta-analysis of randomized controlled trials found diets with legumes resulted in better weight loss (−1.74 kg) and weight maintenance (−0.29 kg) compared to control diets [26]. However, as demonstrated in our current study, a disadvantage of legume-based meals is the lower palatability. This could be an obstacle for increasing the intake of vegetable protein in the population, especially in the Western world where legumes are not part of a traditional diet. 

Our findings should be interpreted while considering the following. The test meals did not represent a typical Danish breakfast, which could have influenced energy intake, subjective appetite sensations, and palatability ratings. The participants were all males and were constrained to the laboratory which reduces the generalizability of the results. However, we chose only to include men to obtain a homogenous group, and for practical reasons, as the time point where women are tested in their menstrual cycle may affect energy intake (higher energy intake during the luteal compared to the follicular phase) [27]. A further limitation is the inclusion of both normal weight and overweight participants. 

## 5. Conclusions

In conclusion, iso-caloric, macronutrient-balanced, fiber-matched meals based on vegetable protein from fava beans and split peas or animal protein from veal and pork or eggs, were found to have similar effects on ad libitum energy intake and subjective appetite sensations. Also, a similar meal based on eggs without supplemented fiber did not differ from the fiber-matched meals.

## Figures and Tables

**Table 1:** Timeline of the measurements on each test day.

Time from Baseline (Minutes)												
	−5	0	15	30	60	90	120	150	180	~200	~200 to ~920	
Test meal		x										
Ad libitum meal									x			
Subjective appetite sensations	x		x	x	x	x	x	x	x	x		
Palatability			x							x		
Gastro-intestinal feelings									x			
Compensatory eating											x	

**Table 2:** Recipes and macronutrient composition of the four test meals.

Meat/Fiber (g)		Bean/Pea (g)		Egg/Fiber (g)		Egg (g)	
Pork/veal 4%	135	Fava Beans	100	Egg, whole	211	Egg, whole	211
Onion	10	Split peas	90	Egg white	40	Egg white	40
Potato	264	Onion	10	Onion	10	Onion	10
Potato flour	38.5	Water	445.5	Potato	305	Potato	338
Water	164	Rapeseed oil	10	Potato flour	41	Potato flour	50
Pea fibers	26	Butter	17	Water	17	Water	0
Rapeseed oil	9	Wheat flour	4.5	Pea fibers	26.5	Cream, 9%	33
Butter	13.2	Vinegar	5	Cream, 9%	33	Tomato ketchup	20
Rasp	20	Tomato ketchup	20	Tomato ketchup	20	Parsley	4
Tomato ketchup	20	Parsley	4	Parsley	4	Garlic	2
Parsley	4	Garlic	2	Garlic	2	Salt	3.3
Garlic	2	Salt	3.3	Salt	3.3	Pepper	0.02
Salt	3.3	Pepper	0.02	Pepper	0.02		
Pepper	0.02						
Energy (kJ)	3564.8	Energy (kJ)	3567.1	Energy (kJ)	3565.6	Energy (kJ)	3569.0
Weight (g)	709	Weight (g)	711	Weight (g)	713	Weight (g)	711
Density (kJ/g)	5.0	Density (kJ/g)	5.0	Density (kJ/g)	5.0	Density (kJ/g)	5.0
Protein (E%)	18.5	Protein (E%)	18.7	Protein (E%)	18.9	Protein (E%)	18.7
CHO (E%)	53.5	CHO (E%)	53.3	CHO (E%)	53.0	CHO (E%)	53.3
Fat (E%)	28.0	Fat (E%)	28.0	Fat (E%)	28.1	Fat (E%)	28.0
Fiber (g)	28.8	Fiber (g)	28.4	Fiber (g)	28.5	Fiber (g)	6.0

E%: energy percent; kJ: kilojoule; CHO: carbohydrate.

**Table 3:** Baseline characteristic of the participants.

	Mean ± SD	Range
Age (year)	26.5 ± 5.5	19–39
BMI, kg/m^2^	23.3 ± 1.9	22.2–27.8
Restraint ^1^	5.67 ± 2.87	0–11
Disinhibition ^1^	5.03 ± 2.12	2–13
Hunger ^1^	4.11 ± 2.57	1–11

^1^ Based on the three-factor eating questionnaire [12]. SD, standard deviation; BMI, body mass index.

## Supplementary Materials

The following are available online at www.mdpi.com/2072-6643/10/1/96/s1, Table S1: Mean differences between the four test meals in subjective appetite sensations, well-being, palatability, and compensatory eating; Figure S1: The four test meals.
