# Supplementary material for: Protein from Meat or Vegetable Sources in Meals Matched for Fiber Content has Similar Effects on Subjective Appetite Sensations and Energy Intake—A Randomized Acute Cross-Over Meal Test Study
Source: Nutrients. 2018 Jan 16;10(1):96. doi: 10.3390/nu10010096 (PMC5793324; doi:10.3390/nu10010096)
Supplement: Supplementary file 1 [file nutrients-10-00096-s001.pdf]

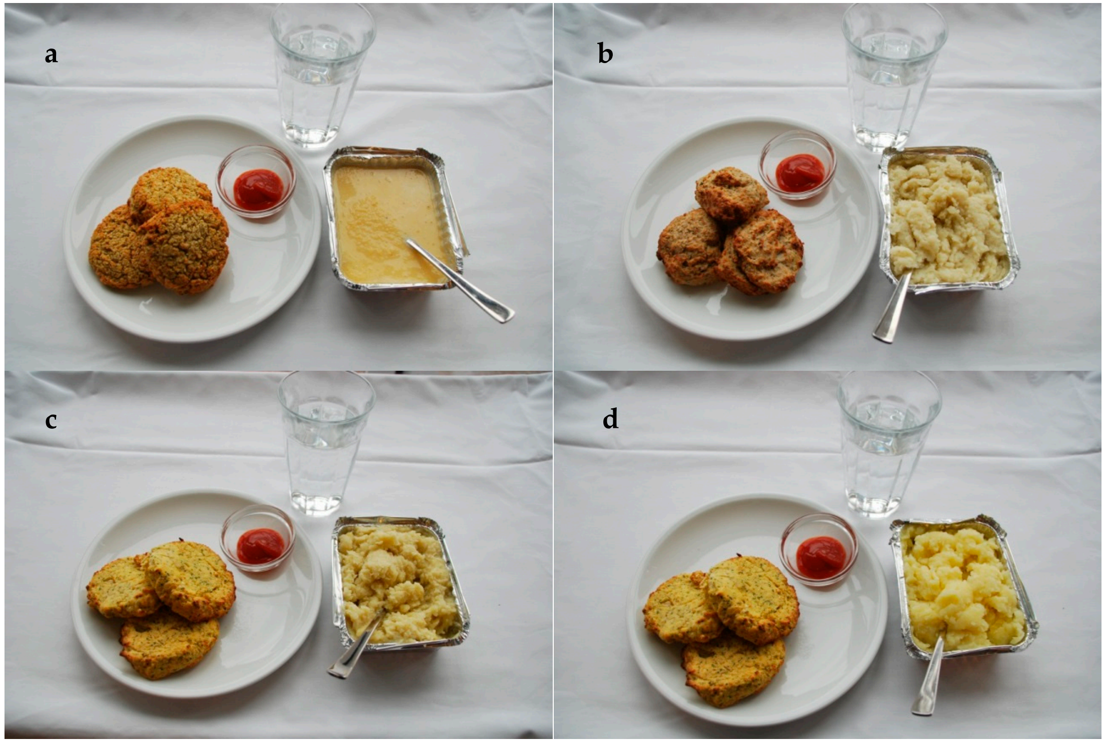

Figure S1. The four test meals. **a**) Patties with beans served with mashed split peas, **b**) Patties with veal/pork and pea fibers served with mashed potatoes with pea fibers, **c**) Patties with eggs and mashed potato, **d**) patties with eggs and pea fibers served with mashed potato with pea fibers.

Table S1. Mean differences between the four test meals in subjective appetite sensations, well-being, palatability and compensatory eating.

	Time-meal interaction	Meal effect	Egg-meat/fiber		Bean/pea-meat/fiber		Egg/fiber–meat/fiber		Bean/pea-egg		Egg/fiber-egg		Egg/fiber–bean/pea	
	p	*p*	Mean diff.	*p*	Mean diff.	*p*	Mean diff.	*p*	Mean diff.	*p*	Mean diff.	*p*	Mean diff.	*p*
Energy intake (kJ)	-	0.13	135.3 ± 111.1	0.62	-28.2 ± 111.4	0.99	-122.8 ± 111.2	0.69	-163.5 ± 110.3	0.45	-258.1 ± 107.6	0.08	-95.6 ± 108.9	0.82
Satiety														
Fasting (mm)	-	0.23	-4.5 ± 3.8	0.63	2.7 ± 3.8	0.89	1.0 ± 3.7	0.99	7.2 ± 3.8	0.219	5.5 ± 3.7	0.44	-1. 7 ± 3.7	0.97
180 min (mm)	0.68	0.14	-1.2 ± 2.0	0.93	-0.2 ± 1.9	1.0	3.0 ± 1.9	0.40	1.0 ± 2.0	0.95	4.2 ± 1.9	0.12	3.2 ± 1.9	0.34
iAUC	-	0.46	507 ± 662	0.87	-440 ± 664	0.91	342 ± 662	0.96	-946 ± 660	0.48	-165 ± 645	0.99	781 ± 651	0.63
Hunger														
Fasting (mm)	-	0.12	0.6 ± 3.8	1.0	-6.4 ± 3.8	0.35	-5.7 ± 3.8	0.44	-7.0 ± 3.8	0.266	-6.3 ± 3.7	0.33	0.6 ±3.8	1.0
180 min (mm)	0.43	0.13	-0.6 ±1.7	0.99	0.4 ± 1.73	1.0	-3.1 ± 1.7	0.26	0.9 ± 1.7	0.95	-2.6 ± 1.7	0.42	-3.5 ± 1.7	0.16
iAOC	-	0.34	331 ± 617	0.95	-761 ± 619	0.61	-175 ± 617	0.99	-1092 ± 618	0.29	-505 ± 604	0.84	586 ±608	0.77
Fullness														
Fasting (mm)	-	0.21	2.7 ± 3.4	0.86	1.8 ± 3.4	0.95	7.1 ± 3.4	0.16	-0.9 ± 3.4	0.99	4.4 ± 3.3	0.56	5.2 ± 3.4	0.40
180 min (mm)	0.88	0.24	-2.0 ± 2.6	0.87	1.1 ± 2.6	0.98	3.1 ± 2.6	0.62	3.0 ± 2.6	0.65	5.2 ± 2.6	0.18	2.1 ±2.6	0.84
iAUC	-	0.71	-654 ± 706	0.79	30 ± 708	1.0	-385 ± 705	0.95	684 ± 704	0.77	268 ± 688	0.98	-416 ± 694	0.93
PFC														
Fasting (mm)	-	0.12	1.6 ± 2.6	0.92	-2.6 ± 2.6	0.74	-4.0 ± 2.6	0.40	-4.2 ± 2.5	0.35	-5.6 ± 2.5	0.11	-1.4 ± 2.5	0.94
180 min (mm)	0.59	0.37	0.6 ± 2.1	1.0	-1.4 ± 2.1	0.90	-2.8 ±2.1	0.54	-2.0 ± 2.1	0.77	-3.4 ± 2.1	0.36	-1.36 ± 2.1	0.91
iAOC	-	0.94	230 ± 496	0.97	-60 ± 497	1.0	83 ± 496	1.0	-290 ± 493	0.94	147 ± 481	1.0	144 ± 486	1.0
Composite score														
Fasting (mm)	-	0.17	-0.9 ± 2.9	0.99	3.42 ± 2.9	0.63	4.5 ±2.9	0.40	4.37 ± 2.9	0.42	5.45 ± 2.8	0.21	1.1 ± 2.8	0.38
180 min (mm)	0.76	0.23	-0.9 ± 2.1	0.97	0.1 ± 2.1	0.97	2.9 ± 2.1	0.51	1.0 ± 2.1	0.96	3.8 ± 2.0	0.26	2.75 ± 2.0	0.54
iAUC	-	0.85	49 ± 542	1.0	-373 ± 543	0.90	-63 ± 442	0.99	-422 ± 540	0.86	112 ± 527	0.99	310 ± 532	0.94
Well-being														
Fasting (mm)	-	0.61	-1.7 ± 2.9	0.93	-4.0 ± 2.9	0.53	-2.3 ± 2.9	0.87	-2.2 ± 2.9	0.87	-0.5 ± 2.9	1.0	1.7 ± 2.9	0.93
180 min (mm)	0.58	0.08	0.51 ± 2.5	0.99	-4.7 ± 2.5	0.22	-3.7 ± 2.5	0.42	-5.2 ± 2.5	0.15	-4.3 ± 2.4	0.29	1.0 ± 2.5	0.98
iAUC	-	0.77	341 ± 418	0.85	-55 ± 418	1.0	178 ± 417	0.97	-396 ± 416	0.78	-163 ± 406	0.98	233 ± 410	0.94
Palatability														
Test meal (mm)	-	<0.001	6.7 ± 4.6	0.47	21.5 ± 4.7	<0.001	15.5 ± 4.7	0.005	14.8 ± 4.7	0.008	8.8 ± 4.5	0.21	-6.0 ± 4.6	0.55
Ad libitum meal (mm)	-	0.09	-1.4 ± 2.1	0.91	1.9 ± 2.14	0.87	-3.3 ± 2.1	0.40	3.3 ± 2.1	0.40	-1.9 ±2.1	0.79	-5.2 ± 2.1	0.06
Compensatory eating	-	0.75	-323 ± 505	0.92	-326 ± 506	0.92	-525 ± 504	0.72	-3.27 ± 504	1.0	-203 ± 493	0.98	-199 ± 497	0.98

iAOC: incremental area over the curve; iAUC: incremental area under the curve; PFC: prospective food consumption. Data are presented as mean difference ± SE.
